# Thriving Between Work and Care in Hong Kong: Associations of Work-family Enrichment with Depression

**DOI:** 10.1093/geroni/igaf122.4335

**Published:** 2025-12-31

**Authors:** Bobo Lau, Crystal Ng, Crystal Ying Chan, Steve Fu-Fai Fong, Florence Fong, Doris S F Yu, Rebecca Choy-Yung

**Affiliations:** Hong Kong Shue Yan University, Hong Kong, China (People’s Republic); Hong Kong Shue Yan University, Hong Kong, China (People’s Republic); The Chinese University of Hong Kong, Hong Kong, China (People’s Republic); Hong Kong Shue Yan University, Hong Kong, China (People’s Republic); Lingnan University, Hong Kong, China (People’s Republic); The Chinese University of Hong Kong, Hong Kong, China (People’s Republic); Golden Age Foundation, Hong Kong, China (People’s Republic)

## Abstract

Six out of ten American family caregivers are employed. In Hong Kong, up to one-third of the over 1 million family caregivers for older adults and adults with disabilities are in full-time employment. Work-family enrichment postulates that people gain happiness, knowledge, and tangible resources from one role (e.g., caregiving) and may be transferred to benefit another role (e.g., work), challenging the notion that roles compete for resources and are often conflictual. Between May and July 2025, this online survey collected data from 353 Hong Kong Chinese adults providing care to an adult relative and working full- or part-time (72.0% female; mean age = 46.1 (SD = 11.4)). It explored the impact of work-family enrichment domains on caregiving burden, gains and depression. Based on the Patient-Health Questionnaire-9, 21.8% and 14.7% of the sample showed moderate and severe depressive symptoms respectively. Path analysis tested the mediating roles of burden and gains between six enrichment domains and depression. Two indirect effects were significant: (i) work-to-family development enhances gain and suppresses depression (95% C.I. = -0.78 to -0.17); (ii) transfer of positive mood from family to work reduces burden and depression (95% C. I. = -1.43 to -0.13). The model explained 45% of depression’s variance. Work-family enrichment works bidirectionally (family to work and work to family) and both affectively and cognitively. With an example from a family-friendly social enterprise, we will discuss how employers may facilitate social capital and benefit-finding in employees’ dual role, besides adjustable work hours and shifts, for effective support.

